# Editorial: Nature Inspired Protective Agents Against Oxidative Stress

**DOI:** 10.3389/fphar.2022.859549

**Published:** 2022-02-11

**Authors:** Manuela Oliverio, Stefania Bulotta, Noélia Duarte

**Affiliations:** ^1^ Dipartimento di Scienze della Salute, Università Magna Græcia di Catanzaro, Catanzaro, Italy; ^2^ Research Institute for Medicines (iMed.Ulisboa), Facultade de Farmacia, Universidade de Lisboa, Lisbon, Portugal

**Keywords:** antioxidants, oxidative stress, mithocondrial dysfunction, neurodegeneration, cardiovascular disease

Oxidative stress is the result of an imbalance between pro-oxidant and antioxidant species at cellular level, also defined as a lack in redox signaling and control. Despite small amounts of reactive oxygen or nitrogen species (ROS and RNS) are essential to maintain cell homeostasis and redox signaling, currently, it is well understood that chronic oxidative stress conditions are responsible for several key biomolecule modifications, such as, DNA impairment, lipid peroxidation and protein carbonylation (Pisoschi et al., 2021). Ultimately, oxidative stress is tightly implicated in the pathophysiology of chronic disorders, such as diabetes, neurodegenerative and cardiovascular diseases, and cancer (Sharifi-Rad et al., 2020).

Antioxidants are the first line of defense against the injurious effects of pro-oxidant species. By definition, an antioxidant is a molecule that prevents or retards the oxidation of a biomolecule both acting as radical scavengers (primary antioxidants) or modulating cellular mechanisms responsible for reactive species production (secondary antioxidants). They can be classified as enzymatic or non-enzymatic, or according to their mechanism of action, as preventive antioxidants, radical scavengers, repair antioxidants or antioxidants exploiting an adaptation mechanism. Finally, they can also be classified on the basis of their origin as endogenous, such as enzymes or small molecules produced by metabolic routes, and exogenous such as synthetic molecules and plant-derived secondary metabolites, normally not synthesized by human body whose intake is mainly due to diet or dietary supplements (Pisoschi et al., 2021).

Literature describing the chemistry, absorption, and metabolism, mechanism of action and biological involvements of such antioxidants is very extensive, dating back to 1960 (Yeung et al., 2019). Nevertheless, the research on protective agents against oxidative stress never stopped growing, still resulting in a hot topic for the recent literature.

The articles presented in this Research Topic further gathered scientific data and provided experimental evidence, therefore contributing to the potential development of new solutions for antioxidant implementation, development, and integration in therapeutic strategies.

Recent discoveries assessing that oxidative stress is the main risk factor for several diseases without therapy, such as neurodegenerative diseases (NDs i.e., Alzheimer disease) and autoimmune disorders (i.e., multiple sclerosis, lupus, rheumatoid arthritis), opened new potential therapeutic approaches for their modulation. In addition, recent studies disclosing the involvement of oxidative stress in mitochondrial damage (Bobadilla et al., 2021; Mannucci et al., 2021), open new molecular pathways where antioxidants can play key roles.

**FIGURE 1:**
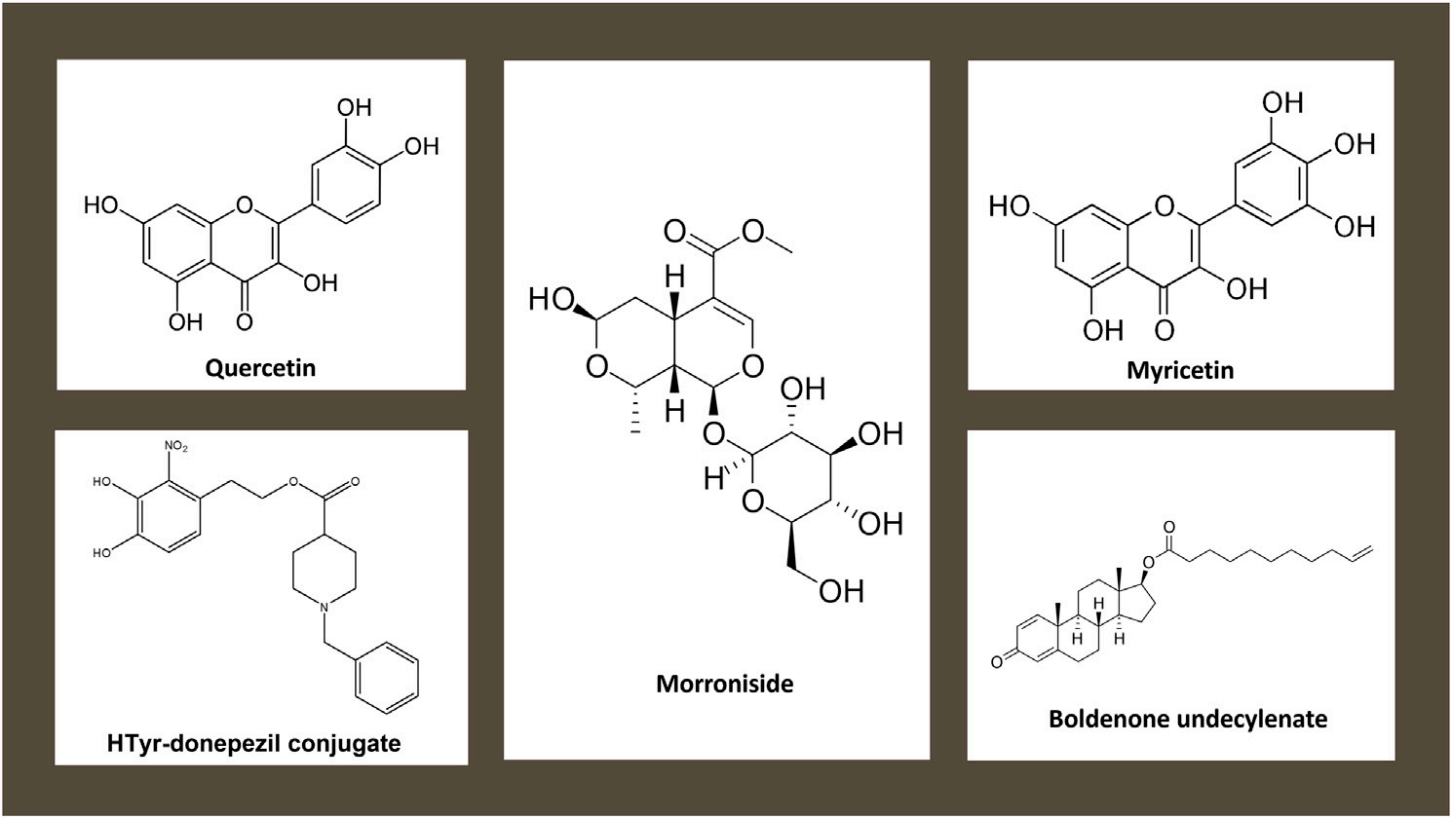
Chemical structures of natural and nature inspired antioxidant molecules investigated by this research topic.

In this context, Costanzo et al., developed novel hydroxytyrosol-donepezil hybrids (Figure 1), and characterized their ROS scavenging, metal-chelating, and cytotoxic properties *in vitro* and in neuroblastoma cells. Among the donepezil hybrids, nitro hybrid HT2 and homovanillyl hybrid HT3a showed the most interesting antioxidant effects. The nitro hybrid HT2 also exerted chelating properties against all metal cations. Based on these results, the authors state that the nitro hybrid HT2 could be a potential lead compound for the treatment of NDs.

Recent evidences indicate the secoiridoid glycoside morroniside (Figure 1) as a small molecule non-peptide glucagon-like peptide-1 receptor (GLP-1R) agonist, suggesting it as optional treatment strategy against ischemic stroke. Liu et al. presented a study supporting the neuroimmunodulatory effect of morroniside, by exploring microglial activation both *in vitro* and *in vivo*. The authors proved that morroniside induced M2 microglia polarization and stimulated IL-10 expression specifically in cortical primary microglia by p38β MAPK pathway. The molecule protected nerve cells against H_2_O_2_-induced cell oxidative damage. In addition, the secoiridoid glycoside blocked ischemic injury by reducing infarct size in mice, an effect in part mediated by enhanced expression of microglial IL-10 in the cortical penumbra area.

Oxidative stress and mitochondrial dysfunction are involved in mechanisms of cardiac toxicity induced by aluminum phosphide (AlP). In his study, Salimi et al. showed that the flavonoid myricetin (Figure 1) can ameliorate AIP-induced cardiotoxicity in rat heart isolated cardiomyocytes. This is achieved by decreasing ROS production and maintaining the mitochondrial membrane potential.

Quercetin (Figure 1) has been reported to have a large variety of biological activities, mainly attributed to its antioxidant properties. With their data, Zhou et al. supported the protective role of antioxidant quercetin on microcystin (MC)-LR toxicity, a cyclic heptapeptide cyanotoxin, on male reproductive system in experimental *in vivo* model. Quercetin mitigated the MC-LR toxic effects by protecting tight junction destruction via the inhibition of oxidative stress and the p-Akt signaling pathway in Sertoli cells.

Boldenone undecylenate (BLD, Figure 1) is a widely known anabolic–androgen steroids (AASs) that leads to increased levels of oxidative stress markers in several organs as kidney and liver. Behairy et al. aimed at exploring the ability of vitamin C to mitigate hepatorenal damage caused by BLD, using an *in vivo* model. It was concluded that vitamin C oral supplementation was able to significantly reduce BLD-induced hepatorenal complications in co-treated rats, probably by reducing lipid peroxidation and modifying the antioxidant protection system.

Despite the promising results in pre-clinical studies, the safety and efficiency of antioxidants in clinical therapy is still under debate, thus resulting in a translational gap needing more unbiased investigations on complex metabolic mechanisms involved in human. Indeed, few clinical studies characterized by statistically significant patients’ number have been reported yet and, in most cases, their conclusions did not support the positive data obtained in pre-clinical studies (Bartekovà et al., 2021; Asbaghi et al., 2021).

Therefore, modern research on antioxidant is expected to address, besides the discovery of new antioxidant field of action, also the translational gap between pre-clinical and clinical antioxidant therapies.
